# A Facile and Template-Free One-Pot Synthesis of Mn_3_O_4_ Nanostructures as Electrochemical Supercapacitors

**DOI:** 10.1007/s40820-015-0074-0

**Published:** 2015-11-13

**Authors:** Zhenjun Qi, Adnan Younis, Dewei Chu, Sean Li

**Affiliations:** School of Materials Science and Engineering, University of New South Wales, Sydney, NSW 2052 Australia

**Keywords:** Electrochemical deposition, Supercapacitors, Charge storage

## Abstract

In this paper, we report an effective, simple, and cost-effective strategy of electrochemical deposition to prepare hausmannite Mn_3_O_4_ thin films for the applications of supercapacitors. Various precursor concentrations and deposition durations were manipulated to tailor the surface morphologies of Mn_3_O_4_ nanostructures and to optimize their electrochemical performances. The Mn_3_O_4_ samples prepared at 0.05 M Mn(NO_3_)_2_ solution for 30 min delivered a large gravimetric specific capacitance of 210 F g^−1^ at a current density of 0.5 A g^−1^, and a good rate capability over other samples. This superior electrochemical performance may be attributed to the improved electrode conductivity with increased accessible area for electrolytes ions. Furthermore, a nanocomposite film based on Mn_3_O_4_/carbon foam was fabricated by utilizing the developed optimized conditions. The Mn_3_O_4_/carbon foam films exhibit an excellent specific capacitance with negligible degradation in retaining specific capacitance values up to 4000 cycles. These findings could further broaden the applications of hausmannite Mn_3_O_4_ in electrochemical energy storage electrodes.

**Electronic supplementary material:**

The online version of this article (doi:10.1007/s40820-015-0074-0) contains supplementary material, which is available to authorized users.

## Introduction

With the diminishing supply of traditional fossil fuels and increasing global warming conditions, enormous efforts have been made to search sustainable and renewable energy resources. Besides, new technologies were also exploited to fulfill the demand of future energy storage and conversion devices [1, 2]. Therefore, energy storage devices (e.g., rechargeable batteries, conventional capacitors, and supercapacitors) as an intermediate step to energy conversion have gained growing interests. Among them, supercapacitors with a high power density, high dynamic charge propagation, long cycle life, and low cost are thought to provide a versatile and convenient solution to various emerging energy applications [3–5].

Depending on different storage principles, supercapacitors can be divided into two categories: double-layer capacitors (EDLCs) and pseudocapacitors (or redox supercapacitors). The former relies on electrostatic charge storage at the interface between electrode and electrolyte, which are usually composed of carbon based materials. The latter class of supercapacitors is based on a fast and reversible redox reaction to store charge and is mostly prominent in transition metal oxides and conducting polymers [1, 5, 6]. To date, RuO_2_ is believed to be the most outstanding material for high electrochemical performance. However, its high cost and toxicity restrict its large-scale industrial applications [7, 8]. Therefore, the exploration of inexpensive and eco-friendly transition metal oxides to replace RuO_2_ for electrochemical energy storage applications is a topic of great debate.

Hausmannite (Mn_3_O_4_) is considered as one of the promising electrode materials due to its environment friendly nature, low cost, natural abundance, good stability, and high theoretical specific capacitance (~1400 F g^−1^) [9–11]. However, it also exhibits poor electrical conductivity (about 10^−7^–10^−8^ S cm^−1^) [12], which severally restricts its practical applications as high-performance electrode materials for supercapacitors. One of the common approaches to overcome this intrinsic drawback of poor conductivity is to structure it on a highly conductive substrate, such as various carbon materials and Ni foam [13]. For example, the Mn_3_O_4_/Ni foam composites were constructed to achieve a specific capacitance of 263 F g^−1^ at a current density of 1 A g^−1^ [14], which was over 10 times higher than that of the Mn_3_O_4_/Ni plate. This phenomenon shows that highly conductive substrate and three-dimensional (3D) substrate could provide fast ion and electron transfer rate, large surface area and good conductivity. The nanostructures of MnO_2_/Mn_3_O_4_ with high surface area are considered as an alternative approach to improve the conductivity of the electrodes.

In this paper, we take the advantages from the afore mentioned two approaches to first fabricate manganese oxide nanostructured and then deposit the as-prepared materials onto highly porous and conductive carbon foam to enhance its capacitance capabilities. The hausmannite Mn_3_O_4_ was first fabricated on Fluorine-doped Tin Oxide (FTO) glass substrate, and the effects of different precursor concentrations and deposition times on the morphology and electrochemical performances were measured and optimized. Subsequently, the hausmannite Mn_3_O_4_/Carbon foam nanocomposite was fabricated which demonstrated a very high charge storage capacity with tremendous cyclic rate capability over 4000 cycles. Such a strategy could also effectively be utilized in designing many other metal oxides/carbon based materials as promising supercapacitor electrode materials.

## Experimental

All chemicals used in the experiments were analytical grade and purchased from Sigma Aldrich without further purification. The Mn_3_O_4_ thin films were carried out using electrochemical deposition process in 100 mL Mn(NO_3_)_2_ aqueous solution with a constant current (1.25 mA) at 70 °C by using an electrochemical workstation (Autolab 302N Potentiostat). A standard three-electrode setup in an undivided cell was used. FTO and carbon foam were used as the working electrodes, while platinum foil (0.2 × 10 × 20 mm^3^) was used as the counter electrode. The distance between the two electrodes was 30 mm. The reference electrode was an Ag/AgCl electrode in 3 M KCl solution, against which all the potentials reported herein were measured. Various concentrations of Mn(NO_3_)_2_ solution (0.01, 0.05, and 0.1 M) were used to deposit films for 30 and 60 min, respectively. The as-prepared samples were dried at room temperature overnight. For convenience, the Mn_3_O_4_ samples with concentrations of 0.01, 0.05, and 0.1 M deposited for 30 and 60 min are re-named as Mn(0.01@30), Mn(0.05@30), Mn(0.1@30), Mn(0.01@60), Mn(0.05@60), and Mn(0.1@60) respectively. The mass of each electrode is presented in Table 1 of Supporting Information.

The phase composition of as-prepared samples was studied by X-ray diffraction (XRD, PANalytical Empyrean with Cu Kα). The morphologies and microstructures of the samples were studied by scanning electron microscopy (SEM, Nova Nano SEM 230) and transmission electron microscopy (TEM, Philips CM200). The electrochemical properties including cyclic voltammetry (CV) and Galvanostatic charge–discharge measurements were performed in 3 M Na_2_SO_4_ aqueous solution by using electrochemical work station (Autolab 302N).

## Results and Discussion

**Fig. 1:**
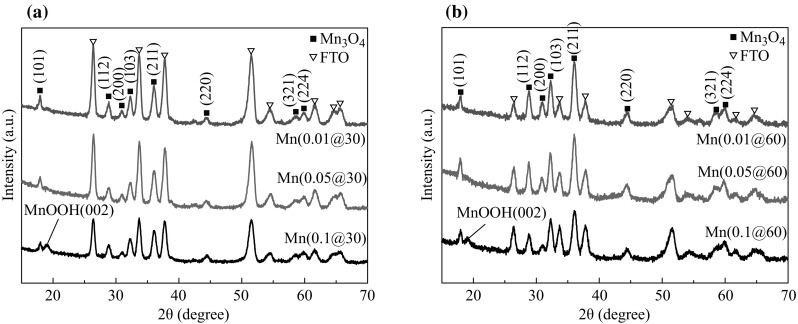
XRD patterns of samples **a** Mn(0.01@30), Mn(0.05@30), Mn(0.1@30); and **b** Mn(0.01@60), Mn(0.05@60), Mn(0.1@60)

The XRD spectra of Mn_3_O_4_ thin films electrodeposited on FTO substrates are shown in Fig. 1a, b. The observed diffraction peaks marked by black block symbols correspond to tetragonal hausmannite manganese oxide structure (JCPDS card no. 04-008-0316). By increasing the molar concentration to 0.1 M, an additional peak, which corresponds to tetragonal manganese oxide hydroxide phase (JCPDS card no. 00-018-0804), was appeared at both electrodeposition times.

The existence of this extra peak may be attributed to the fact that higher precursor concentration results in higher electrodeposition rate, thus leading to the increase local concentration of H^+^ ions which facilitates MnOOH growth [15]. By comparing both figures, it can also be seen that the same diffraction peaks are observed at different deposition time; however, the ratio of diffraction peak intensities between (211) and its neighboring peaks of the FTO increases from 0.75 to 1.3, when the deposition time was prolonged from 30 to 60 min. Therefore, it can be inferred that the deposition time has no considerable influence on the Mn_3_O_4_ film structure other than increasing film thickness.

**Fig. 2:**
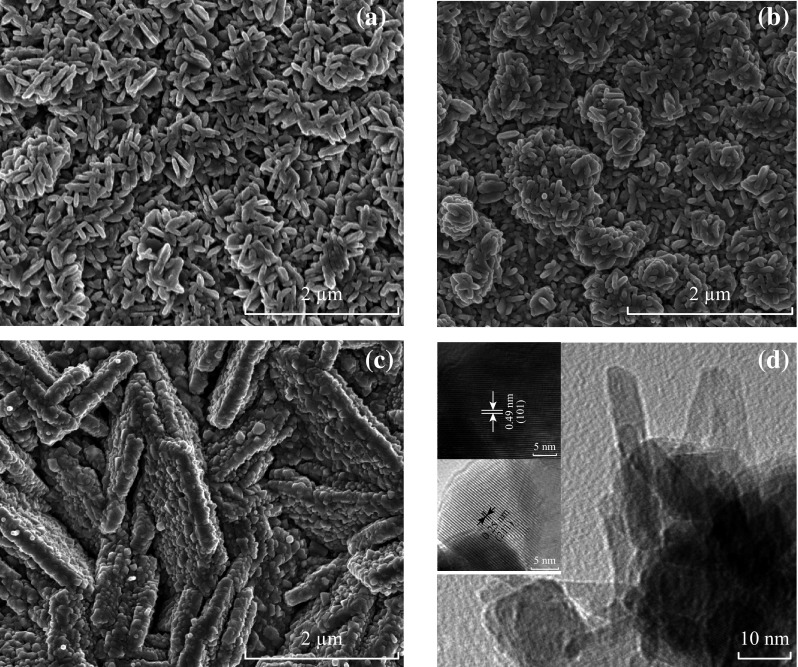
Surface morphologies of Mn_3_O_4_ samples **a** Mn(0.01@30), **b** Mn(0.05@30), and **c** Mn(0.1@30). **d** TEM image of Mn(0.05@30) showing cross-inter-linked nano-platelets with the length range from 20 to 50 nm and the width range from 5 to 10 nm. The inset HRTEM images showing lattice spacing

The surface morphologies of electrochemically deposited Mn_3_O_4_ thin films on FTO substrates [Mn(0.01@30), Mn(0.05@30), Mn(0.1@30)] are shown in Fig. 2a–c. Many cross- or inter-linked tiny Mn_3_O_4_ platelets are randomly distributed throughout the substrate area in the form of loose bunches with nano-micro pores as shown in Fig. 2a. By increasing molar concentration to 0.05 M, the size of Mn_3_O_4_ platelets increased and forms relatively closely packed structure as shown in Fig. 2b. Such a structure could have capability of high capacity charge storage. Further increasing the concentration of Mn(NO_3_)_2_ to 0.1 M, the surface morphology transformed to irregular-shaped micro-sized pillars/walls which are constructed by nano–micro Mn_3_O_4_ particles arranged as brick upon tile pattern as shown in Fig. 2c.

The TEM images were utilized to further explore the microstructures of as-prepared Mn(0.05@30) sample. From Fig. 2d, the cross- and inter-linked nano-platelets can be verified with the length range from 20 to 50 nm and the width range from 5 to 10 nm. The HRTEM images reflect well-resolved lattice fringes, and inter-planar spacing of 0.25 and 0.49 nm corresponds to (211) and (101) planes as illustrated in the inset of Fig. 2d. These values match well with the XRD results, thus verifying the tetragonal structure of hausmannite manganese oxide.

**Fig. 3:**
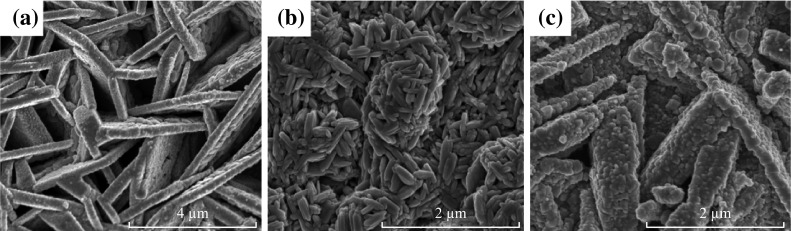
Surface morphologies of Mn_3_O_4_ samples prepared for 60 min **a** Mn(0.01@60), **b** Mn(0.05@60), and **c** Mn(0.1@60)

Figure 3 shows the morphologies of the corresponding samples deposited for 60 min. It can be seen from Fig. 3a, by increasing deposition time, the tiny platelets stuck together in a way to form several interconnected micro-walls. For Mn(0.05@60), the interconnected and tightly packed bunch-like structure of Mn_3_O_4_ platelets can be found as shown in Fig. 3b. However, the longer deposition time (60 min) enlarged the size of Mn_3_O_4_ platelets that may lead to a decreased surface area. Moreover, the additional deposition time also enlarged the size and diameter of the micro-pillars and micro-walls compared to the sample prepared for 30 min as shown in Fig. 3c. These findings further authenticate the XRD results which state that the longer deposition time may only facilitate film thickness.

**Fig. 4:**
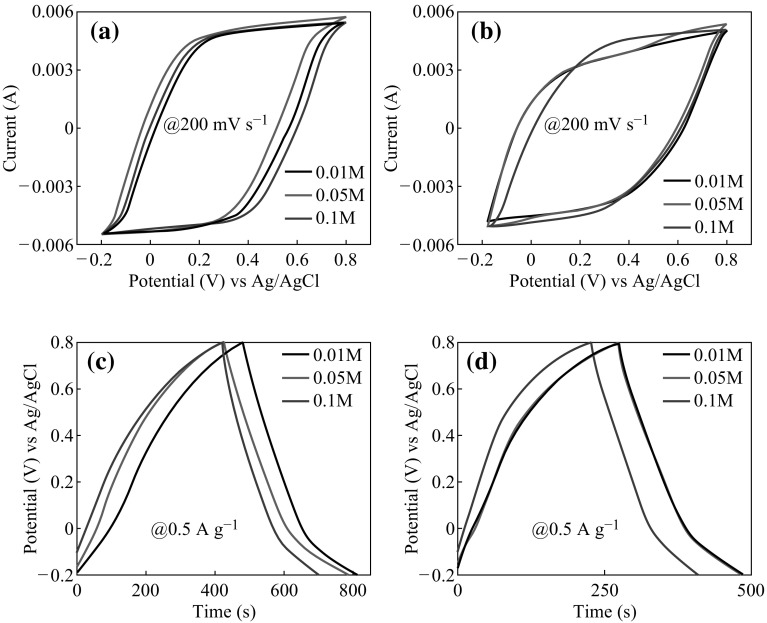
Cyclic voltammetry and Galvanostatic charge–discharge curves: CV curves of **a** Mn(0.01@30), Mn(0.05@30), Mn(0.1@30); **b** Mn(0.01@60), Mn(0.05@60), Mn(0.1@60) at voltage sweep rate of 200 mV s^−1^. The Galvanostatic charge–discharge curves of **c** Mn(0.01@30), Mn(0.05@30), Mn(0.1@30); **d** Mn(0.01@60), Mn(0.05@60), Mn(0.1@60), samples at current density of 0.5 A g^**−1**^

The electrochemical performances of all deposited Mn_3_O_4_@FTO samples were investigated by conducting cyclic voltammetry and Galvanostatic charge–discharge curves in 3 M Na_2_SO_4_ aqueous solution with a three-electrode system. Figure 4a, b shows the CV and Galvanostatic charge–discharge curves for Mn_3_O_4_@FTO electrodes prepared in different concentrations of Mn(NO_3_)_2_ solution at different deposition times at a scan rate of 200 mV s^−1^. All curves exhibit nearly rectangular and symmetrical shapes, exhibiting kinetic reversibility of redox process and good capacitive behavior. The larger loop area of the CV curve for the Mn(0.05@30) sample indicates its superior specific capacitance over other samples. Furthermore, its peak current density remains more stable than that of the other samples in a large potential window. These characteristics demonstrate the superior capacitive performance of the Mn(0.05@30) electrode over other electrodes.

The typical Galvanostatic charge–discharge curves of Mn_3_O_4_@FTO thin films were tested at a constant current density of 0.5 A g^−1^ and in the potential range from −0.2 to 0.8 V as depicted from Fig. 4c, d. The potential window is chosen to avoid the occurrence of oxygen evolution reaction. The nearly linear and symmetrical charge–discharge curves pointed towards good capacitance performance of all electrodes. However, the charge and discharge times of the Mn(0.05@30) samples are much higher than all other samples. This may be attributed to the fact that Mn(0.05@30) samples have higher specific area than Mn(0.01@30) and Mn(0.1@30) as confirmed from SEM images (Fig. 2a–c).

Moreover, the longer deposition time enlarged the particles size and subsequently increases the film thickness which directly affect the specific area and hence on charge–discharge times, respectively. The evolution of impurity phases (MnOOH) in Mn(0.1@30) and Mn(0.1@60) samples (low specific capacitance for MnOOH ~ 100 F g^−1^ [16] ) may be an additional justification for their lower charge–discharge times and poor charge storage capacities over Mn(0.05@30/60).

**Fig. 5:**
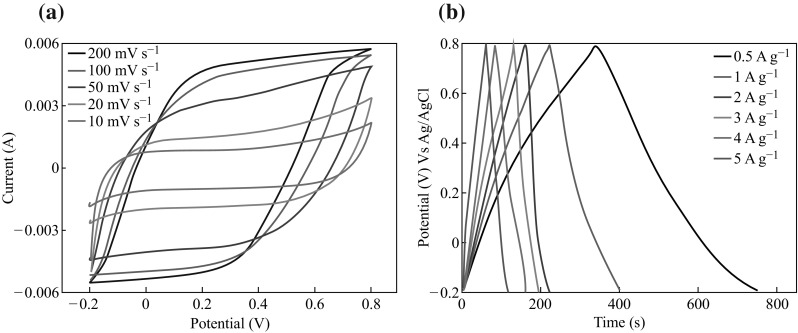
**a** Cyclic voltammetry curves of Mn(0.05@30) electrode at different voltage scan rates; **b** Galvanostatic charge–discharge curves of Mn(0.05@30) electrode at various current densities

The electrochemical properties of Mn(0.05@30) were further examined by taking the CV measurements at different scan rates from 10 to 200 mV s^−1^, as shown in Fig. 5a. It is noteworthy to mention here that the capacitance values can be change/modified with increase in scan rates. As the scan rate increases, electric charges might have some difficulty to occupy the available sites at electrode/electrolyte interface due to their limited rate of migration and orientation in the electrolyte. Also, at high scan rate, when high current is delivered by supercapacitor, a considerable voltage loss (Δ*V*) can be originated which affects the overall capacitance of supercapacitors. The charge/discharge curves of Mn(0.05@30) at various current densities ranging from 0.5 to 5 A g^−1^ can be depicted from Fig. 5b. At high current, considerable voltage loss occurs due to the resistive nature of the device. Also, by increasing charging current, charging process of Mn_3_O_4_ will be difficult due to the limited migration of ions in the electrode [17]. The specific capacitance values of Mn(0.05@30) at various current densities can be found in Table 2 of supporting information. The gradual decrease in discharging time with the increase of current density may be attributed to the low utilization efficiency of active materials under high discharge current.

**Fig. 6:**
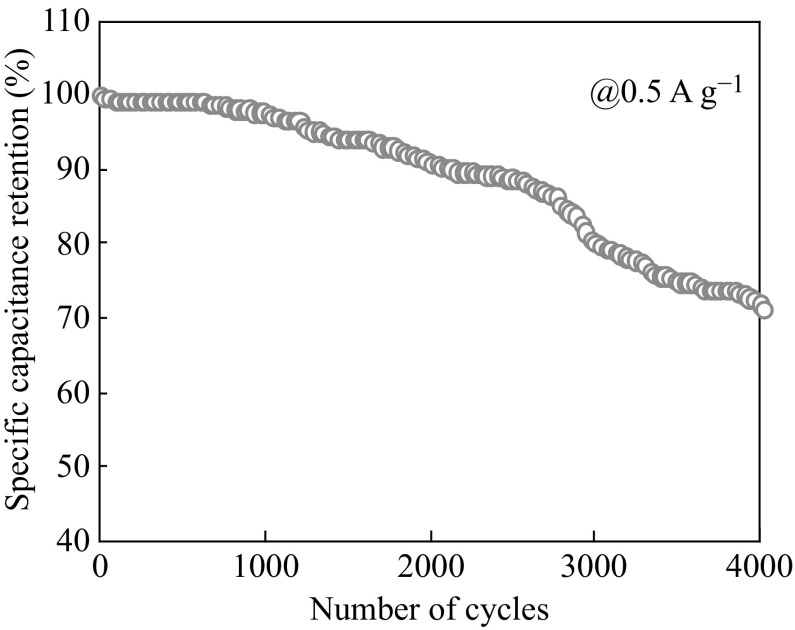
The plot illustrating the retention of specific capacitance during 4000 extensive charge discharge cycles

We ascribe this to the discrepant insertion–de-insertion behaviors of alkali ion (Na^+^) from the electrolyte to Mn_3_O_4_. At a low current density (0.5 A g^−1^), the diffusion of ions from the electrolyte can gain access to almost all of the available pores of the electrode, leading to a complete insertion reaction resulting in high specific capacitance [13]. However, the effective interaction between the ions and the electrode is greatly reduced by increasing current density, and as a result, a reduction in capacitance values can be expected. Moreover, the Mn(0.05@30) supercapacitor demonstrated excellent cycling stability in retaining its specific capacitance at current density of 5 A g^−1^ for up to 4000 cycles as shown in Fig. 6.

The electrode retained about 95 % of its capacitance during first 1000 cycles and showed minimal degradation (up to 70 %) in its specific capacitance retention even after 4000 cycles as shown in Fig. 6. Such impressive cyclic performance of Mn(0.05@30) points towards its great potential as high-performance energy storage electrode.

A number of approaches have been attempted to optimize the electrodes for the enhancement of the specific capacitance. One of the effective approaches is to develop the three-dimensional (3D) hierarchical porous nanostructure. This not only provides a continuous electron pathway but also facilitates ion transportation by shortening diffusion pathways [18, 19]. For example, including nanocomposite device composed of CeO_2_ nanoparticles/graphene was fabricated to increase its performance as a supercapacitor [18]. Moreover, MnO_2_ nano-flowers onto carbon nanotubes [19], NiO/Carbon nanocomposite [20], and Vanadium nitride/CNT [21] composites with excellent performance were previously presented. We also fabricated Mn(0.05@30)/carbon foam composite to evaluate its charge storage performance.

**Fig. 7:**
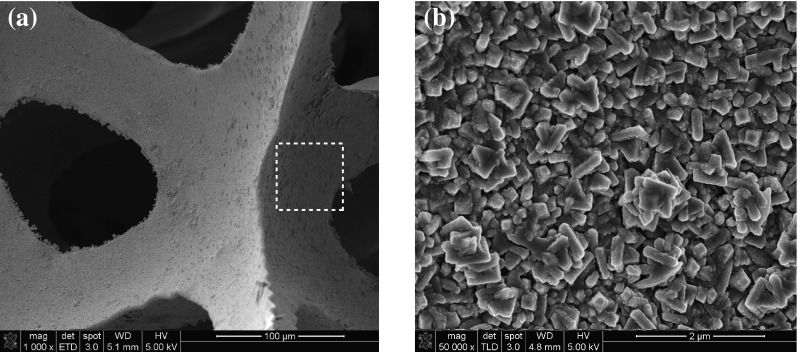
SEM images of Mn(0.05@30)/carbon foam nanocomposite **a** low magnification and **b** high magnification

The surface morphologies of Mn(0.05@30)/carbon foam nanocomposite in both low and high magnification are shown in Fig. 7. It can be seen that tiny Mn_3_O_4_ platelets are transformed into tiny bars which are interconnected to each other and randomly distributed on the surface of carbon foam. Contrary to Mn(0.05@30)@FTO, the structure of Mn(0.05@30)/carbon foam is loosely packed which can be attributed to the porous structure of carbon foam which may provide larger surface area to grow Mn_3_O_4_ nano-platelets. Another possible reason may be the difference in surface energies between FTO and carbon foam 1.18 mJ m^−2^ [22] which greatly influence the surface morphologies of Mn_3_O_4_ at the same concentration and deposition period.

**Fig. 8:**
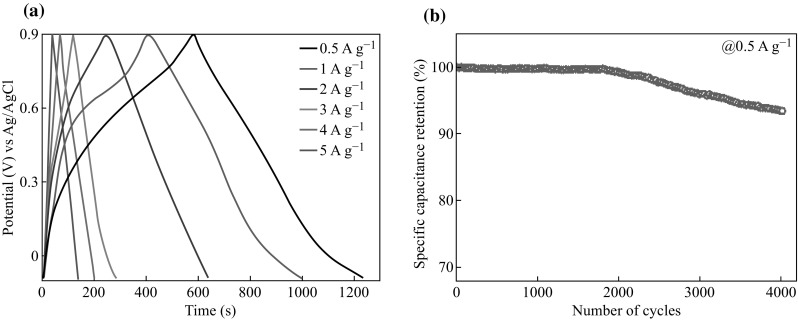
**a** Galvanostatic charge–discharge curves of Mn(0.05@30)/carbon foam at different current densities; **b** Specific capacitance retention test of Mn(0.05@30)/carbon foam nanocomposite for 4000 cycles

The electrochemical measurements of Mn(0.05@30)/carbon foam were carried out in 3 M Na_2_SO_4_ aqueous solution. The nanocomposite device demonstrated much better charge/discharge performances than Mn(0.05@30)@FTO as shown in Fig. 8a. This phenomenon may originate from difference in electrical conductivities between the FTO and carbon foam. The electrical conductivity of carbon foam [23] is about two orders of magnitudes higher than FTO [24], which can supply more space for electrolyte ions and electrons to take part in the faradaic reactions [25]. The Mn(0.05@30)/carbon foam nanocomposite also demonstrated excellent stability by retaining its 99 % of specific capacitance for first 2000 cycles and ends up with 93 % of its specific capacitance retention for up to 4000 cycles as illustrated in Fig. 8b. Such a cyclic retention performance of Mn(0.05@30)/carbon foam is far superior than its counterpart Mn(0.05@30)@FTO which only retain 70 % of its specific capacitance during 4000 cycles. It is worthy of note that the background capacitance of the carbon foam was negligible (Fig. S1), suggesting that the measured capacitance was mainly due to the Mn_3_O_4_–carbon foam nanocomposite. Various factors such as the electrode materials, the appropriate operating voltage, as well as the right selection of electrolyte could be responsible to attain such an impressive cycling stability of the asymmetric supercapacitors.

In addition, the component of carbon foam in Mn(0.05@30)/carbon foam nanocomposite can effectively prevent the partial dissolution of Mn_3_O_4_ in the electrolyte solution and also enhance the mechanical strength, which can alleviate degradation of specific capacitance value upon cycling [25]. The Mn(0.05@30) coated on carbon foam may also able to accommodate the structural/meso-structural changes induced by the guest ions during the intercalation and de-intercalation process to improve the cycling stability. Furthermore, the excellent charge storage performance of the Mn_3_O_4_–carbon foam electrode is probably due to the positive synergistic effects between the Mn_3_O_4_ and carbon foam [26]. Firstly, the Mn_3_O_4_ tiny bars on the carbon foam could reduce the aggregation of the tiny bars (as can be seen from Fig. 7b, less aggregation than Fig. 2b), making nearly every Mn_3_O_4_ tiny bar accessible for electronic and ionic transport pathways. This results in high double-layer capacitance, and importantly, enhancing the utilization of the active materials and mitigating the internal resistance of the capacitors, thus increasing the specific capacitance value and cycle stability of the composite. Secondly, carbon foam can also provide a highly conductive network for electron transport during the charge and discharge processes. Thirdly, the pores within the Mn_3_O_4_ tiny bars can not only provide more active sites for the intercalation and de-intercalation of Na^+^ species, but also improve the accessibility of the composite to the electrolyte ions and shorten the ion diffusion and migration pathways.

The Mn_3_O_4_–carbon foam nano-composites are expected to make a significant contribution to the advancement of high-performance supercapacitors because of the low cost associated with the precursor and the simplicity in the preparation method. Also, they can serve as promising candidates for other applications such as catalysis and lithium-ion batteries.

## Conclusion

In summary, we have demonstrated a general paradigm for fabricating the Mn_3_O_4_-based nanostructures by implementing a simple and versatile electrochemical approach. Such an approach can be mimicked and put an advantage for electrochemical supercapacitor applications. A loose bunch-like nano-platelet-like structure achieved at Mn(0.05@30) presents a better electrochemical properties. In addition, longer deposition time enlarges the particles sizes, leading to a lower specific capacitance. Furthermore, the effects of two different substrate materials on charge storage capacity of Mn_3_O_4_ electrodes were also studied. Among all investigated materials in this work, Mn(0.05@30)/carbon foam nanocomposite exhibits a remarkable supercapacitive performance with ultrahigh specific capacitance (almost double than its counterpart fabricated on FTO substrate). The porous and highly conductive carbon foam can provide large surface areas for the pseudocapacitive reactions and facilitate the contact between the electrochemical active material and the electrolyte ions. These results therefore imply a promising way to improve and modify the morphology characteristics with significant enhancement of electrochemical performance, therefore possess great potential for application in energy storage devices.

## Electronic supplementary material

Below is the link to the electronic supplementary material.

Supplementary material 1 (PDF 56 kb)
