# Supplementary material for: A Facile and Template-Free One-Pot Synthesis of Mn3O4 Nanostructures as Electrochemical Supercapacitors
Source: Nanomicro Lett. 2015 Nov 13;8(2):165–73. doi: 10.1007/s40820-015-0074-0 (PMC6223675; doi:10.1007/s40820-015-0074-0)
Supplement: Supplementary file 1 — Supplementary material 1 (PDF 56 kb) [file 40820_2015_74_MOESM1_ESM.pdf]

Supporting Information for

A Facile and Template-Free One Pot Synthesis of Mn_3O_4 Nanostructures as Electrochemical Supercapacitors

Zhenjun Qi[1], Adnan Younis[1,]*, Dewei Chu*, Sean Li

School of Materials Science and Engineering, University of New South Wales, Sydney, 2052, NSW, Australia

[1]Equal Contribution

*Corresponding authors. E-mail: a.younis@unsw.edu.au, d.chu@unsw.edu.au

Table 1 The mass of different Mn_3O_4 samples

Sample-Name	Mass in (mg)
**Mn(0.01@30)**	0.88
**Mn(0.05@30)**	1.21
**Mn(0.1@30)**	0.89
**Mn(0.01@60)**	1.06
**Mn(0.05@60)**	0.94
**Mn(0.1@60)**	1.13
**Mn(0.05@30)/carbon foam**	1.10

Table 2 The variation in specific capacitance values of Mn(0.05@30) with different current densities

Current Density (A g^{-1})	Specific Capacitance (F g^{-1})
0.5	210
1.0	185
2.0	154
3.0	131
4.0	104.58
5.0	86.43

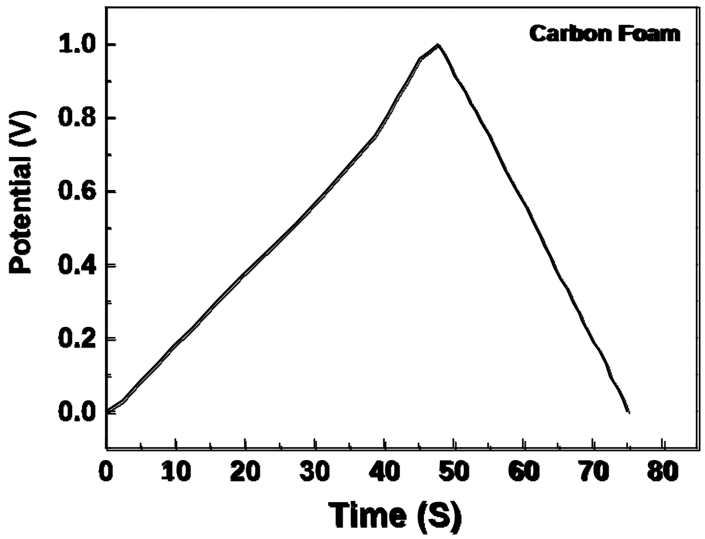

Fig. S1 Charge-discharge curve of pristine carbon foam
